# A new prediction model integrated serum lipid profile for patients with multiple myeloma

**DOI:** 10.7150/jca.69321

**Published:** 2022-03-14

**Authors:** Huizhong Wang, Biyun Chen, Ruonan Shao, Wenjian Liu, Lang Xiong, Li Li, Yue Lu

**Affiliations:** 1 Sun Yat-sen University Cancer Center, Guangzhou, China.; 2 State Key Laboratory of Oncology in South China, Guangzhou, China.; 3 Collaborative Innovation Center for Cancer Medicine, Guangzhou, China.

**Keywords:** multiple myeloma, lipid profile, metabolism, prognostic model

## Abstract

**Purpose:** This study aimed to explore a predictive risk-stratification model combing clinical characteristics and lipid profiles in multiple myeloma (MM) patients.

**Methods:** The data of 275 patients in Sun Yat-Sen University Cancer Center were retrospectively analyzed and randomly divided into the training (n = 138) and validation (n=137) cohorts. Triglyceride (TG), low-density lipoprotein (LDL), high-density lipoprotein (HDL), lactate dehydrogenase (LDH), Apolipoprotein B (Apo B) and Apo B/Apolipoprotein A1 (Apo A1) ratio were the prognostic factors identified through univariate and multivariate Cox analysis.

**Results:** A 6-prognostic factor model was constructed based on Lasso regression. Patients were divided into low- and high-risk groups and the former group showed longer overall survival (OS) time (p<0.05). The area under the curve (AUC) of the risk score model for 5-and 10-year OS were 0.756 [95% CI: 0.661-0.850] and 0.940 [95% CI: 0.883-0.997], which exhibited better accuracy than International Staging System (ISS) and Durie and Salmon (DS) stage.

**Conclusion:** This study aims to combine the lipid metabolism profile with the clinical characteristics of MM patients to generate a prognostic model. The nomogram integrating ISS stage and risk score increased the prediction accuracy. This model can monitor lipid profile as a simple and effective method, which has certain clinical significance for improving the accuracy of the prognosis and exploring potential therapeutic targets.

## Introduction

Multiple myeloma (MM) is a cytogenetically heterogeneous plasma cell clonal proliferative disease that accounts for around 17% of all hematological malignancies 1. Despite the continuous emergence of more effective treatments, the survival time for patients with MM varies from several months to more than ten years 2. The highly variable prognosis for patients with MM suggests that accurate and individualized risk assessments are important to inform treatment decisions and predict survival rates.

Several current prognostic evaluation systems have been widely used in the clinic. The Durie and Salmon (DS) system was first proposed in 1975 3 and includes many parameters and subjective factors that limit its prognostic value since quantitative osteolytic lesions subjective bias in the system and less accurate 4. Subsequently, the International Staging System (ISS) based on albumin (ALB) and β2-microglobulin (β2-MG) level was proposed as a simple and effective prognostic stratification method that is widely used in the clinic 5. The Revised ISS (R-ISS) has added high-risk factors that are known to affect patient prognosis such as abnormal cytogenetics which is a more valuable prognostic tool for patient stratification and in guiding treatment decisions compared to the ISS system 6. However, these tools remain suboptimal and there remains a need for improved prognostic methods in MM including tumor and host-related factors. Also, the accuracy of original prognostic scores can be improved to identify high-risk groups in a more timely and convenient manner. These approaches have a high potential to lead to the development of individualized treatment plans for MM patients.

Recent studies have indicated that the levels of serum lipids play an important role in tumor development and metastasis and have prognostic value 7,8. Studies suggest that changes in tumor lipids also occur in many types of cancer patients 9,10. Lipid profiles are composed of lipids and apolipoproteins molecules and their derivative indices which are clinically distinct and targetable for therapeutic interventions 11,12. Lipid metabolism disorders are important characteristics of cancer cells that are considered potential targets for cancer therapy 13. To date, few studies have focused on the relationship between the levels of serum lipids and prognosis in patients with MM 14,15. The correlation between the lipid content and ratios, the prognosis of MM patients and treatment responses suggests that lipid profiles may have potential clinical significance both in judgment and decision making.

In this study, we aimed to clarify the relationships between lipid and apolipoprotein profiles and the types and stages of MM. We retrospectively analyzed the lipid and apolipoprotein profiles of patients with MM and explored their predictive values when combined with existing prognostic indicators and clinical pathology. We developed a model based on lipid profiles that has high prognostic accuracy. Our data support the inclusion of lipid to improve the predictive accuracy of clinically established biomarkers in the prognosis of MM.

## Materials and methods

### Patient selection

The study was a retrospectively one, approved by the Clinical Research Ethics Committee of Sun Yat-sen University Cancer Center. We analyzed the clinical data of 372 MM adult patients (age ≥18 years) who were pathologically diagnosed and previously untreated in the Sun Yat-sen University Cancer Center (SYSUCC) between January 2007 and September 2019. The exclusion criteria were described as following: a) patients diagnosed with undefined significance, such as smoldering multiple myeloma, primary amyloidosis or solitary extramedullary plasmacytoma, b) patients previously taken or regular intake of any lipid regulating agent, without normal liver and kidney functions, c) patients lacking of data concerning lipid profile data, d) patients without complete follow-up data or died within a month, e) patients with earlier or simultaneously diagnosed other malignant diseases. Finally, a total of 275 patients were eligible for analysis in this study. And the patients were then randomly divided into the training (n = 138) and validation (n = 137) cohorts.

### Treatments and response evaluation

The classical drugs-based regimens given to patients included VAD (vincristine, doxorubicin, and dexamethasone), DVD (vincristine, liposomal doxorubicin, and dexamethasone) 16; bortezomib-based regimens such as PAD (bortezomib, doxorubicin, and dexamethasone) 17 and VTD (bortezomib, thalidomide, and dexamethasone) 18. Patients were given at least four cycles of treatment, followed by ASCT (autologous stem cell transplantation) or thalidomide maintenance if eligible. Evaluation and treatment are based on International Myeloma Working Group (IMWG) standards 19.

### Clinical data and serum lipid profiles

The data of each enrolled patient was acquired after diagnosis and before the initiation of treatment. Baseline clinical characteristics involved age, gender, height, weight, albumin (ALB), serum creatinine (SCr), calcium (Ca), lactate dehydrogenase (LDH), β-2 micro globin (β-2 MG), DS stage, ISS stage, date of diagnosis, date of death or last follow-up, along with initial therapy and response of treatment. While the lipid profiles contained apolipoprotein B (Apo B), apolipoprotein A1 (Apo A1), cholesterol (CHO), triglycerides (TG), high-density lipoprotein (HDL) and low-density lipoprotein (LDL). By the weight (in kilograms) divided by height (in meters) squared calculated body mass index (BMI). The laboratory tests were conducted on fresh blood samples collected after overnight fasting in the laboratory of the Sun Yat-san University Cancer Center. The median time interval between blood sampling and the commencement of treatment was 14 days (3-25 days) and the time interval was not associated with survival.

### Follow-up and study endpoints

Patient follow-up was performed by hospital outpatient follow-up or telephone interviews every 6 months for the first 3 years and then every year to determine recurrence or death. The last follow-up date was November 31st, 2020, to confirm the final conditions of recruited patients. The main outcome of the study was overall survival (OS) which was defined as the time interval between the date of diagnosis and date of death for deceased patients or the date of the last follow-up.

### Cut-off values for prognostic biomarkers

The X-tile software (3.6.1)^20^ was used to determine the optimal cutoff values based on OS for each of potential prognostic biomarkers among lipids and apolipoproteins profiles and stratify patients into low- and high-level sub-groups. Univariate and multivariate Cox analyses were used to evaluate the individual prognostic values of the profiles. The optimum cut-off values for all variables were determined using the X-tile software as follows: Age (60 year), BMI (22), ALB (30 g/L), SCr (81 umol/L), Ca (2.5 mmol/L), ApoB (1.1 g/L), ApoA1 (1.05 g/L), ApoB/ ApoA1 (1.09), TG (1.25 mmol/L), LDH (215 U/L), CHO (3.2 mmol/L), LDL (1.9 mmol/L), HDL (0.9 mmol/L) and β-2 MG (5 mg/L).

### Statistical analysis

A one-way ANOVA was used to analyze continuous variables and the Chisq test or Fisher exact test for categorical variables via the SPSS software version 25 (IBM Corporation, Armonk, NY, USA). Continuous factors of the patients, such as demographic distributions, distribution characteristics of the lipids level and the clinical variables were expressed as the mean ± standard deviation. The X-tile software was used to determine the optimal cut-off values for dividing the continuous variables into categorical variables. Univariable and multivariable Cox regression hazard models were employed to identify independent prognostic factors of OS for MM according to their cut-off values. Next, the least absolute shrinkage and selection operator (LASSO) regression analysis was used to explore the best weighting coefficients of these independent prognostic factors. This approach is an established method for the regression of high-dimensional data. Survival analyses were performed using the Kaplan-Meier method. The prognostic accuracy of the model was quantified using time-dependent receiver operating characteristic curves (ROC) and the area under the curve (AUC). The analyses mentioned above were first performed in the training cohort, and then validated in the validation cohort. All statistical tests were two-sided and differences were significant at a *P*-value threshold of < 0.05. R software (version 3.6.3 for Windows, http://www.R-project.org) was used to perform the statistical analyses.

## Results

### Patient selection and clinical characteristics

**Figure 1:**
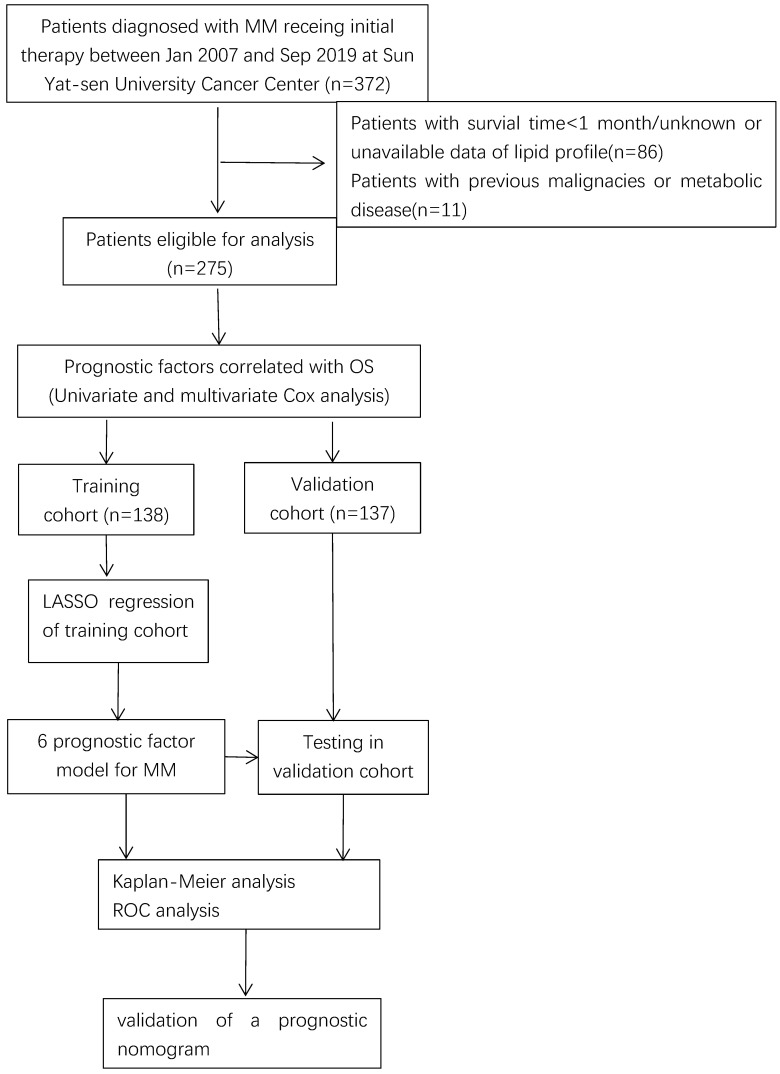
Flow chart of data collection and analysis.

A total number of 275 MM patients were enrolled with available lipids profiles and survival data and randomly divided them into the training cohort (n=138) and validation cohort (n=137) as shown in **Figure 1**. The baseline patient characteristics are summarized in **Supplemental Table 1**. The age of patients ranged from 24 to 84 years with a median age of 60 years. 164 patients (59.6%) were male and 111 (40.4%) patients were female. Approximately half of the patients had a serum monoclonal protein of IgG, while about 22.9% (63) patients with monoclonal protein of IgA and 22.1% (61) patients did not have records of monoclonal protein. In total, 96 (34.9%) patients received bortezomib-based regimens and 179 (65.1%) patients received classical drugs-based regimens. All patients were given at least four cycles of treatment, 18 (6.5%) patients followed by ASCT. Approximately 25% of cases achieved complete response (CR).

### Identification independent prognostic features through survival analysis

**Figure 2:**
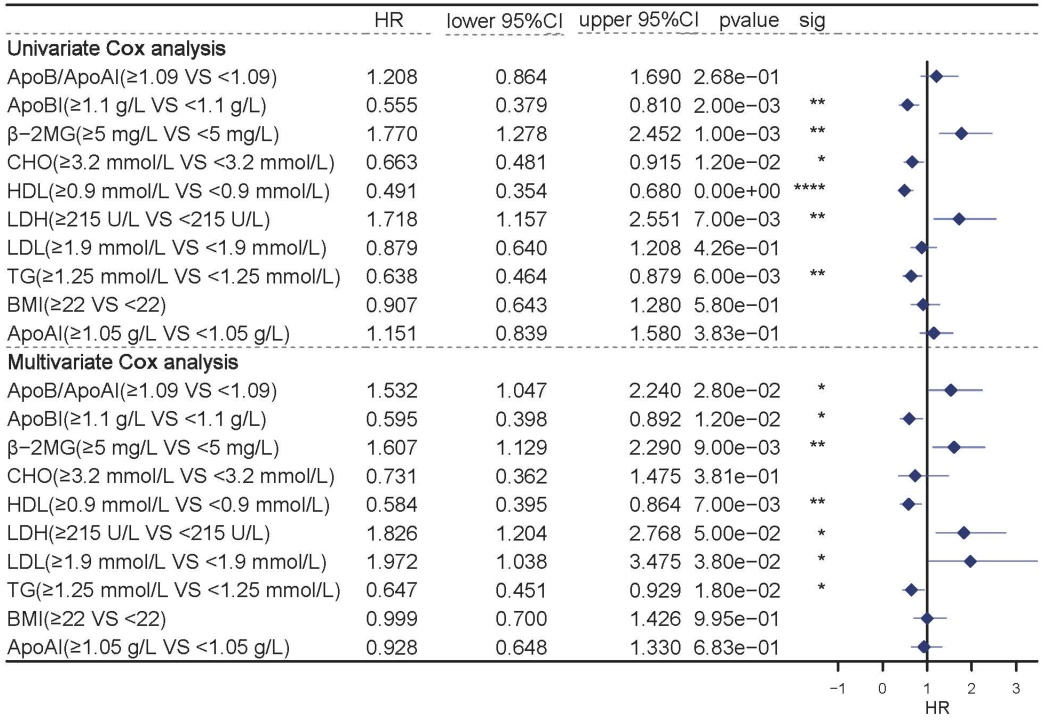
Univariate (top) and Multivariate (bottom) COX analysis in the full group of patients.

The univariate and multivariate COX analyses were used to evaluate the individual prognostic values of the profiles. Univariate Cox analysis showed that lipids including ApoB, CHO, TG, LDH, and HDL were independent prognostic indicators (P<0.05). While multivariate analysis showed that variables including ApoB, TG, LDH, LDL, HDL and the ApoB/ApoA1 ratio were independent prognostic indicators (**Figure 2**, P<0.05).

**Figure 3:**
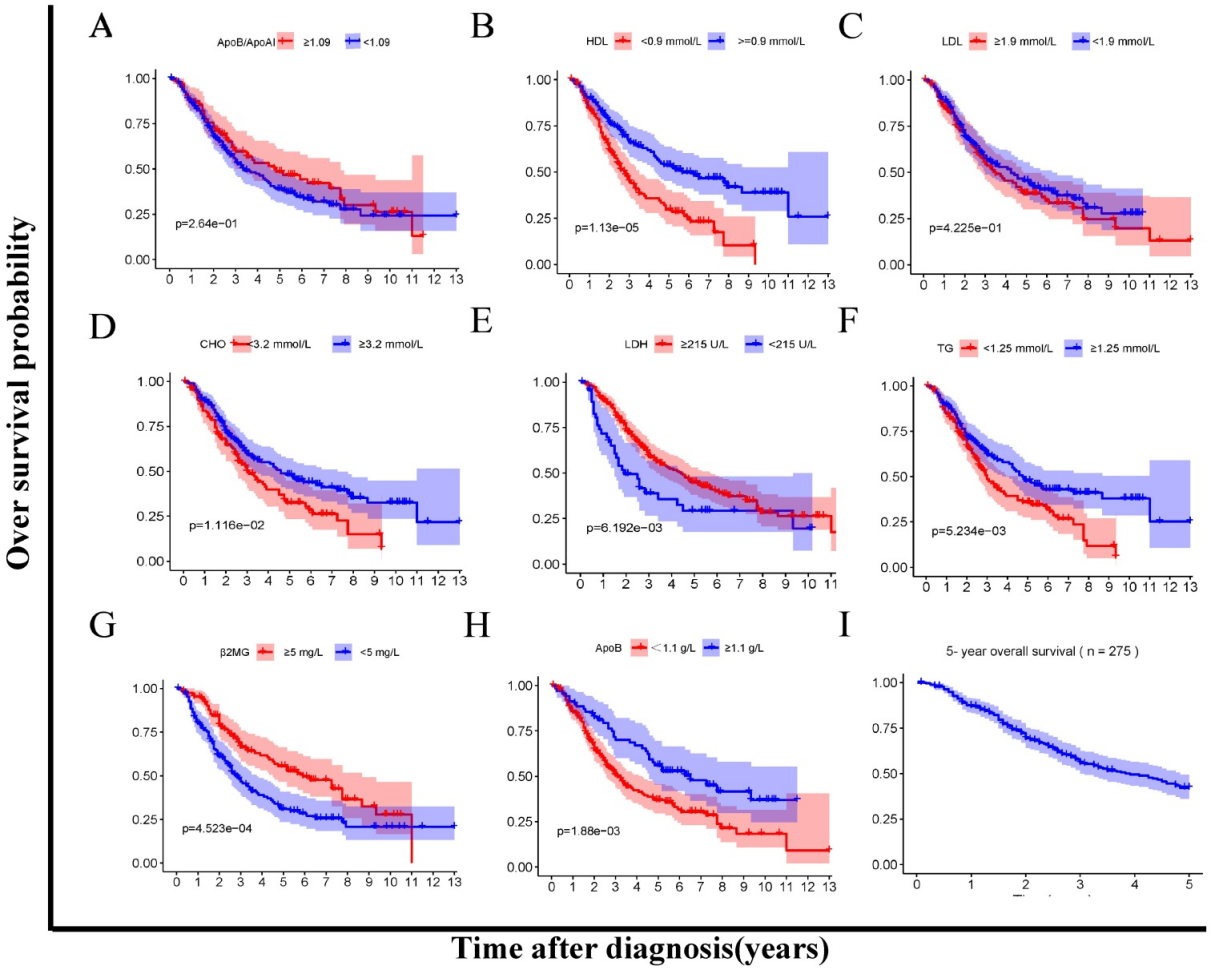
** Kaplan Meier survival analysis between low-and high-level groups of lipid profiles biomarkers (A-H). A.** ApoB/ApoA1 ratio 1.09 < vs ≥1.09. **B.** HDL <0.9 vs ≥ 0.9 mmol/L. **C.** LDL 1.9 <vs ≥ 1.9 mmol/L. **D.** CHO 3.2 < vs ≥ 3.2 mmol/L. **E.** LDH < <215 vs ≥ 215 U/L. **F.** TG <1.25 vs ≥ 1.25 mmol/L. **G.** MG < 5 vs ≥ 5 mg/L. **H.** ApoB < 1.05 vs ≥ 1.05 g/L. I.5-year overall survival.

In addition, we compared the OS between the low- and high- level of the lipids and apolipoproteins with Kaplan-Meier survival analysis respectively (**Figure 3A-H**). The cumulative OS rate of patients in the low HDL, CHO, TG, ApoB group was significantly lower than that of patients in the high group (P<0.05) (**Figure 3B, D, F, H**). Besides, the OS rate of patients in the low LDH, β2MG group was significantly higher than that of patients in the high group (P<0.05) (**Figure 3E, G**).

### Construction and validation of lipid and apolipoprotein risk scores

The potential prognostic factors based on univariate and multivariate Cox analysis were considered to associate with OS. Then, the LASSO regression analysis was employed to construct a prognostic model using the seven prognostic factors (ApoB, TG, LDH, LDL, HDL and ApoB/ApoA1 ratio) in the training cohort. Based on the penalized maximum likelihood estimator of 1000 bootstrap replicates, a 6-prognostic factor model was established via the minimum criteria optimal λ value (**Supplemental Figure 1**). The equation for the model was as follows:

Risk score=-0.75×ApoB serum level + 0.53×ApoB / ApoA1 ratio - 0.28×TG serum level + 0.95×LDH serum level + 0.26×LDL serum level - 0.77×HDL serum level

The lipid profile risk score was generated for each patient according to the above formula. Patients were further grouped into high and low-risk groups according to the median threshold of the lipids profile risk scores based on the training cohort. The distribution of age, ApoB, TG, HDL, LDH, β2MG and ISS stage were significantly different between the two subgroups (**Table 1**).

**Figure 4:**
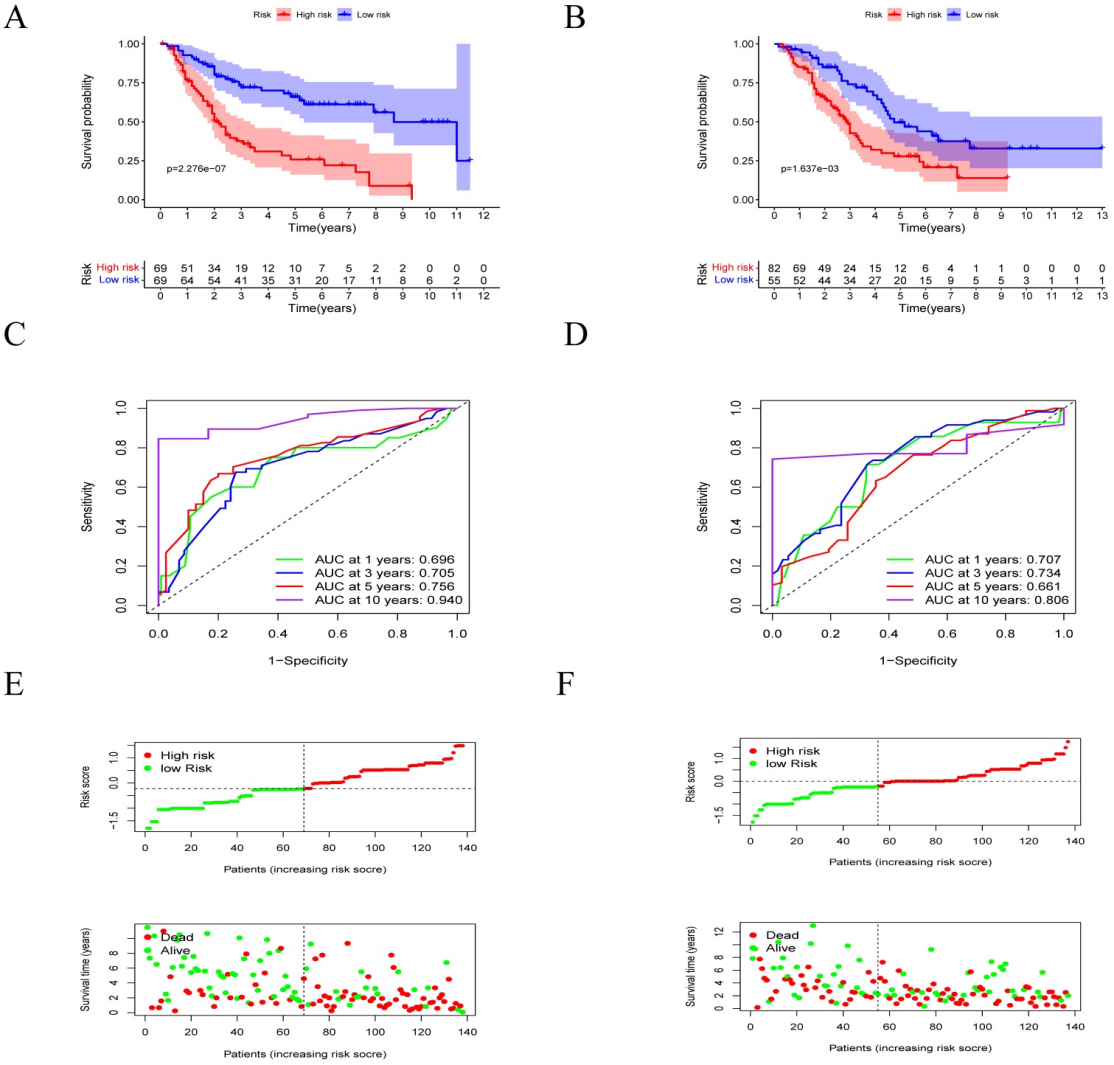
** Construction and validation of the prognostic model. A-B.** Survival difference between high- and low-risk groups in the training cohort and the validation cohorts. **C-D,** Time- dependent ROC analysis for 1- , 3- , 5- and 10- year overall survival (OS) of prognostic model in training the validation cohorts. **E-F.** Risk score analysis of the signature in the high- and low-risk cohorts in the two cohorts mentioned above.

The survival of MM patients is summarized in **Figure 3**. In the low-risk group, patients had a significantly longer OS time compared to the high-risk group (p<0.05) in the training cohort (**Figure 4A**). The AUCs for 1-, 3-, 5- and 10-year survival were 0.696, 0.705, 0.756 and 0.940, respectively (**Figure 4C**). Moreover, it could be seen that treatment response has correlation with risk levels, as shown in **Supplemental Figure 2**. The low-risk group had longer OS time in classical drugs-based regimens and bortezomib-based groups. And the AUCs for 1-, 3-, 5- and 10-year survival showed certain predictive significance.

Furthermore, in validation cohort, the low-risk group had longer OS time (p<0.05) as well (**Figure 4B**). The AUCs for 1-, 3-, 5- and 10-year survival were 0.707, 0.734, 0.661 and 0.806 (**Figure 4D**). We analyzed the distribution of the lipid profile risk scores in patients with different survival outcomes using dot plots to compare the survival of subjects. Our data showed that the survival of patients in the low-risk group was higher than survival in the high-risk group (**Figure 4E-F**) in the two cohorts.

### Univariate and multivariate Cox analysis

**Figure 5:**
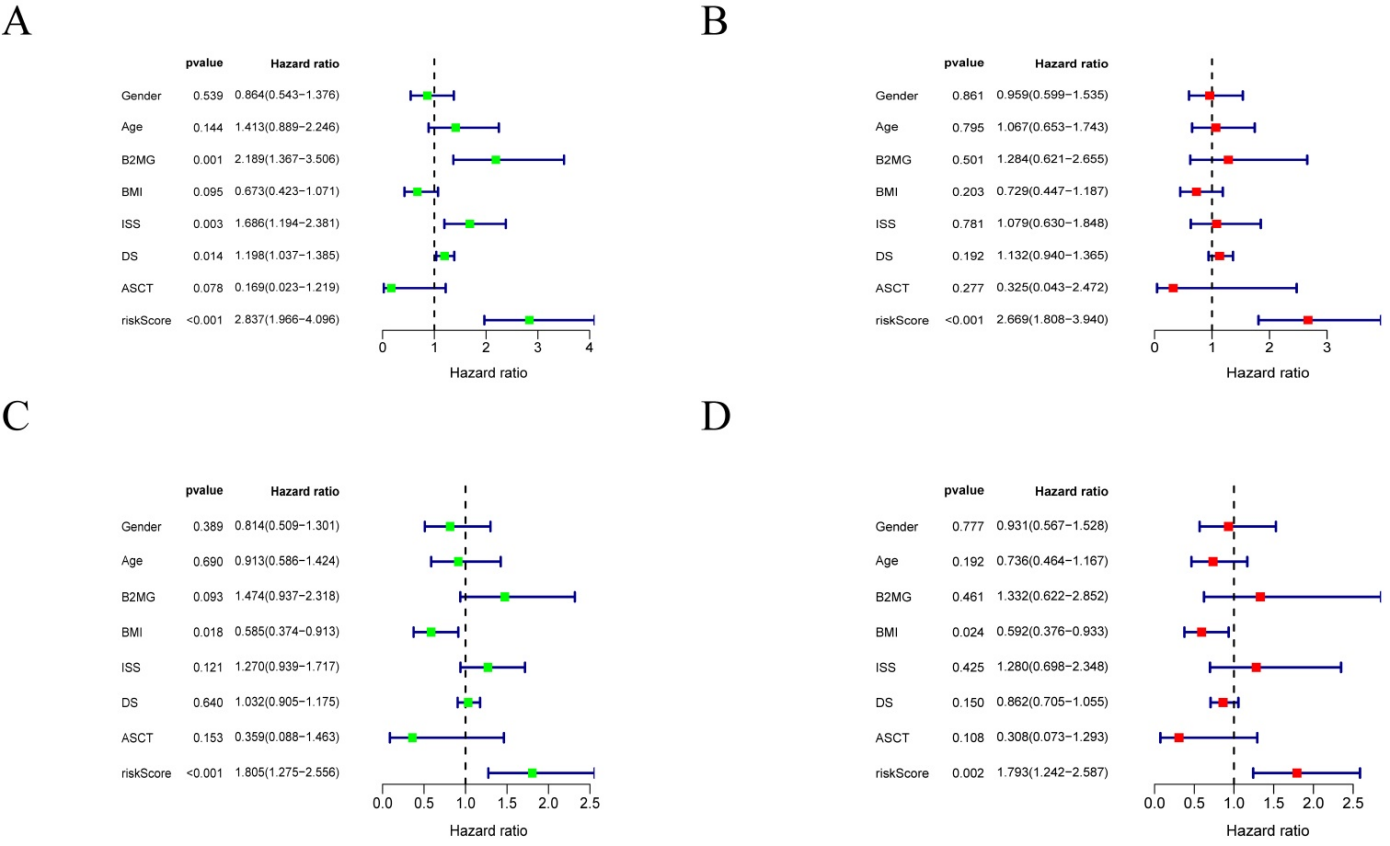
** Forrest plots of the univariate and multivariate Cox analysis in MM. A-B.** Forrest plot of the univariate and multivariate Cox regression analyses in the training cohort. **C-D.** Forrest plot of the univariate and multivariate Cox regression analyses in the validation cohort.

The univariate and multivariate Cox analyses for risk score and other prognostic values were performed in MM. The univariate analysis indicated that the risk score was an independent prognostic indicator for OS (**Figure 5A, C**) in the training and validation cohorts. After adjusting other clinical confounding factors in multivariate Cox analysis, the risk score was still an independent prognostic factor for OS (**Figure 5B, D**) in the two cohorts mentioned above.

### Comparison of the prognostic factors and merged risk scores

**Figure 6:**
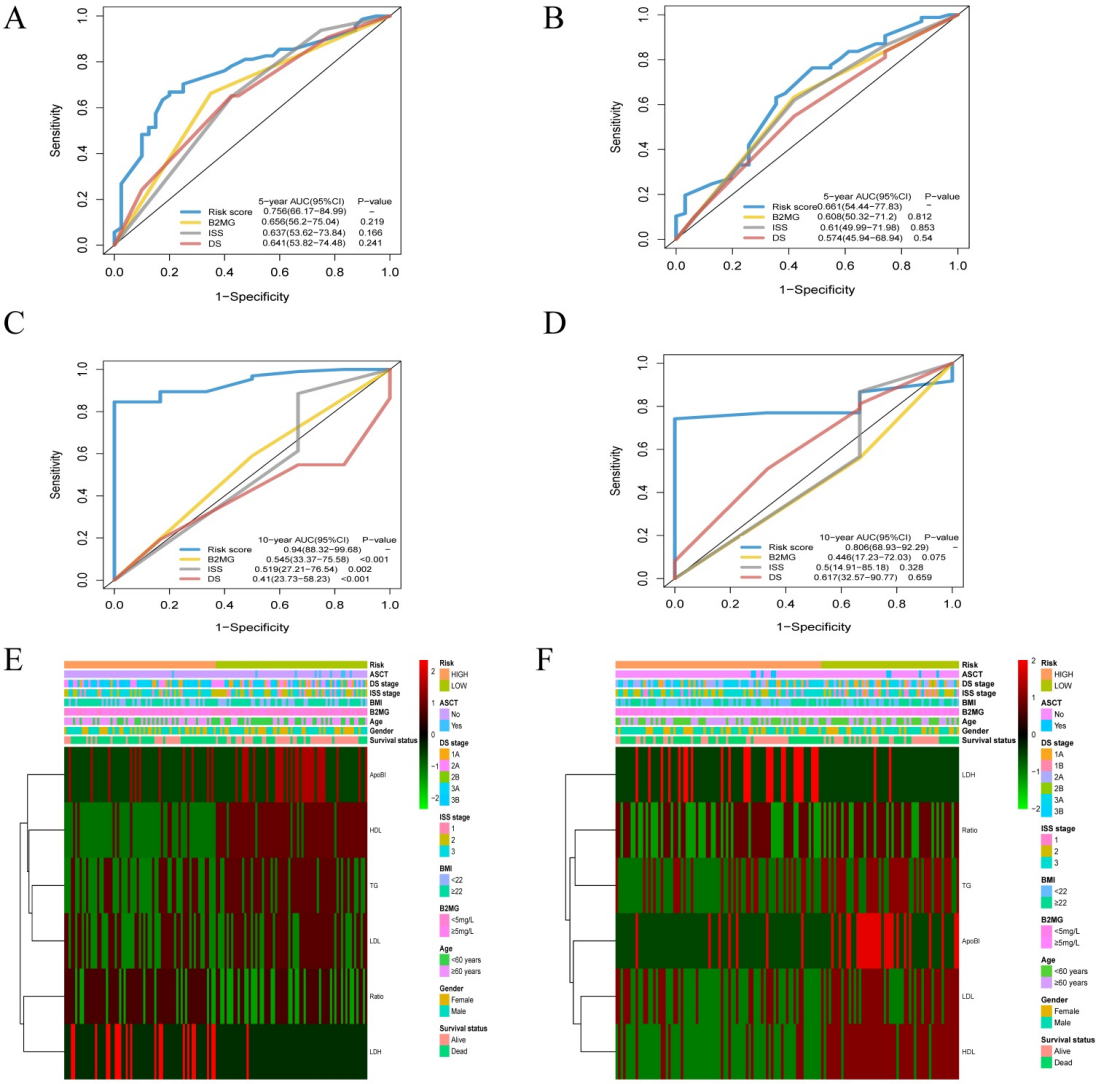
** Time-dependent receiver operating characteristic (ROC) analysis and Heatmap of lipids risk score compared to clinicopathological characteristics in different risk score. A-D.** The AUCs of 5-, and 10-year risk score model has significant difference from that of β-2 MG, the DS and ISS stage model for the training cohort (A, C) and the validation cohort (B, D). **E-F.** Heatmap of the 6-prognostic factor profile and clinicopathological characteristics in different risk score for the training cohort (E) and the validation cohort (F).

The AUC of the risk score model for 5-year OS was 0.756 [95% CI: 0.662-0.850]. The AUCs for the level of β2-MG, ISS and DS stage models were 0.656 [95%CI: 0.562-0.750], 0.637 [95%CI: 0.536-0.738] and 0.641 [95% CI: 0.538-0.745] in the training cohort. However, there were no significant differences in prognostic accuracy was observed between the three models (**Figure 6A**). The AUC of the risk score model for 3-year OS was 0.661 [95% CI: 0.544-0.778]in the validation cohort (**Figure 6B**). The AUC of the risk score model for 10-year OS was 0.940[95% CI: 0.883-0.997] (**Figure 6C**), while the β2-MG, ISS and DS stage models were 0.545 [95%CI: 0.338-0.756], 0.519 [95%CI: 0.272-0.765] and 0.410 [95% CI: 0.273-0.582], which was significantly higher than the AUCs for ISS and DS stage models. The AUC of the risk score model for 10-year OS was 0.806 [95% CI: 0.689-0.923] (**Figure 6D**) in the validation cohorts. Heat maps were used to compare the clinical characteristics and level of serum lipid profiles. Patients with high-risk scores were associated with older age and a higher stage of disease (**Figure 6E-F**).

**Figure 7:**
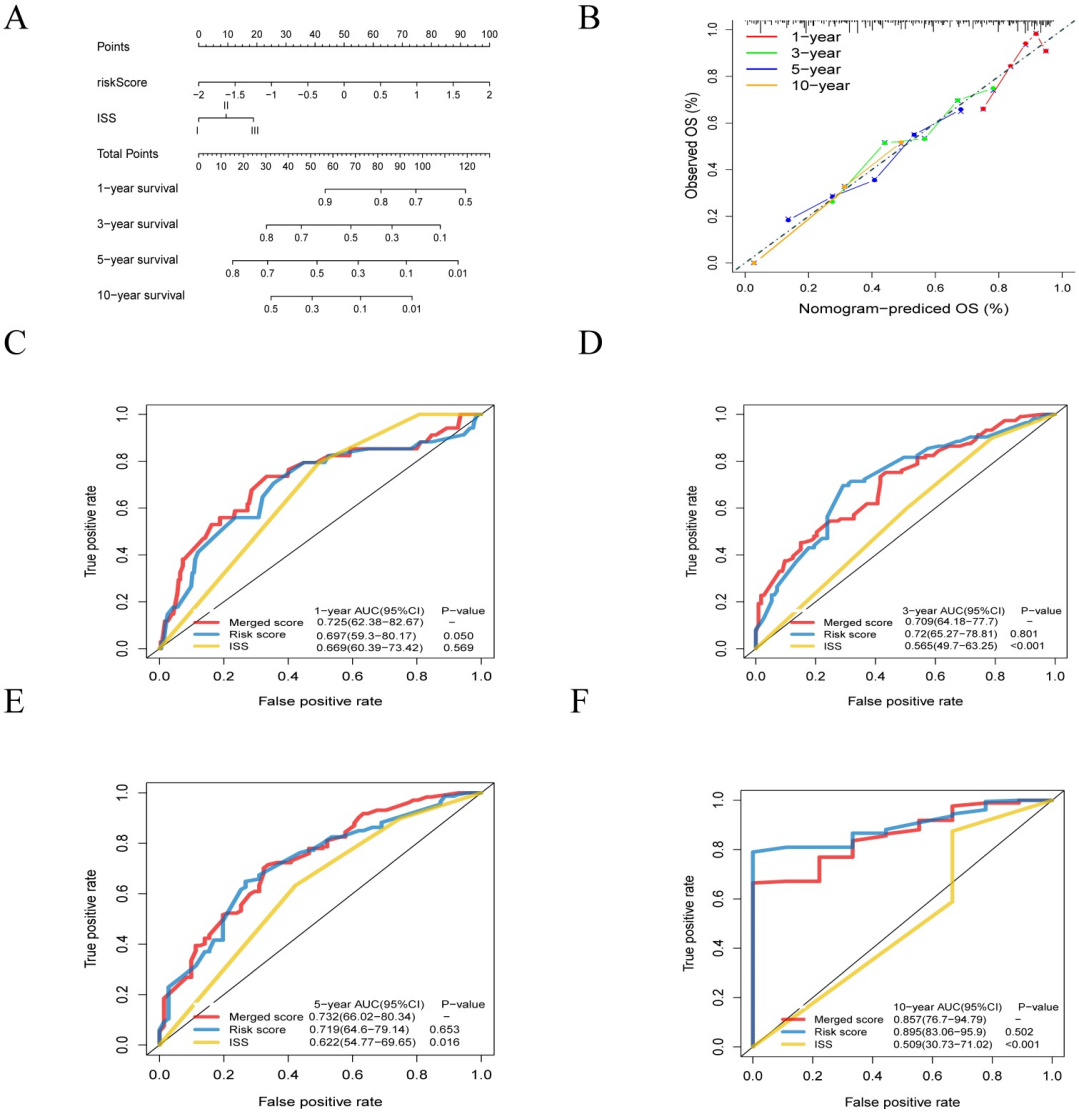
** Building and validation of the nomogram to predict the overall survival of patients with multiple myeloma. A.** Nomogram plot was built based on lipids risk score and ISS stage. **B.** Calibration plot of the nomogram. **C-F.** The AUC of 1- , 3- , 5- and 10-year risk score model was higher than that of the ISS stage.

Furthermore, to generate a more accurate evaluation system, a nomogram was used to integrate the classic prognostic factors, the ISS stage (**Figure 7A**) combining two cohorts. The calibration plots showed good performance of the nomogram in predicting the 1, 3, 5, 10-year OS (**Figure 7B**). The AUC of merged score for 1, 3, 5, 10-year were 0.725 [95% CI: 0.624-0.827], 0.709 [95% CI: 0.642-0.777], 0.732 [95% CI: 0.660-0.803] and 0.857 [95% CI: 0.767-0.948], which was significantly higher than ISS stage, suggesting that the nomogram can enhance the OS prediction compare to the standard prognostic factor (**Figure 7C-F**).

## Discussion

In the present study, lipid profiles were combined with the clinic-pathological characteristics of MM patients to generate a unique prognostic model which demonstrated enhanced prognostic value relative to the standard staging system. This model included six parameters (ApoB, TG, LDH, LDL, HDL and ApoB/ApoA1) and can be used to stratify MM patients into high and low-risk groups that had significant differences in OS. Also, a nomogram was developed to predict survival and the model was tested with the time-dependent ROC curve. Our results indicated that the model had improved prognostic value compared to the other staging systems.

The relationship between lipids and apolipoproteins in MM has not been systematically investigated and few comprehensive studies have reported on the clinical significance of lipid profiles in MM. To the best of our knowledge, no previous study has combined lipids and apolipoproteins as potential prognostic indicators in patients with MM. In this study, we analyzed the lipids and apolipoprotein levels of MM patients using a multi-layered approach. We found that most circulating lipids and apolipoproteins levels in MM patients are related to survival. We also retrospectively analyzed the relationship between risk level and clinicopathological features, treatment and prognosis. In the present study, 179 patients were treated with classical drugs-based regimens and 96 patients received bortezomib-based regimens. It was found that both in two subgroups, patients in low-risk group had longer OS time. Moreover, combined ROC analysis, our model showed it also has certain predictive significance for the survival of high- and low-risk patients in subgroups. In these cases, the lipid profiles are related to the etiology and prognosis of MM. Lipid profiles and several parameters identified as having prognostic value 4,5 were united to build a new model, dividing patients into high-risk and low-risk groups, with a significant difference in OS. While tested with time dependent ROC curve, the model based on lipid and apolipoprotein profiles showed statistical significance compared with the ISS and DS staging system.

In MM, precision medicine approaches are required along with an improved understanding of the role of metabolism in tumorigenesis 13. This knowledge may be used to develop new serum markers that can accurately monitor disease in MM patients. Lipid profiles can be practically used in routine testing as they can be easily analyzed and obtained in a high throughput manner.

ApoB levels directly correlate with circulating serum cholesterol levels which can transport lipids to cells in the human body 21. Apolipoprotein A-1 (ApoA-1) is the main protein component of high-density lipoprotein (HDL) encoded by ApoA-1 which plays an important role in lipid metabolism and inflammation 22,23. Previously, apolipoprotein profiles have been used to predict the development and recurrence of some tumors and have showing diagnostic properties 24-26. In our study, we found that the ApoB and ApoB/ApoA1 ratio are risk factors for MM which is consistent with the previously mentioned study. In some studies, ApoA 11,15,27 has shown low accuracy which may be related to sample size and selectivity bias. Studies have also found that MM patients have hypocholesterolemia with lower levels of cholesterol, LDL-C and HDL-C levels compared to healthy individuals 28,29. We found that MM patients have longer survival times when their total cholesterol and HDL-C levels are higher, which is consistent with the study mentioned above. HDL-C has antioxidant and anti-inflammatory properties and may protect against cancer. Conversely, HDL-C can inhibit myeloma proliferation by reducing the levels of cytokines in granulocyte-monocyte progenitor cells and bone marrow cells.

In this study, we aimed to assess lipid levels and the relationship between these levels and prognosis of patients with MM. Abnormal lipid metabolism is an important feature of tumor cells and is also a potential target for tumor treatment 30,31. Studies have shown that compared with healthy controls, patients with myeloma have abnormal lipid levels 15,28,32. In addition, *in vivo* and *in vitro* studies have found that statins, which are HMG-CoA reductase inhibitors used for the treatment of hyperlipidemia, may increase the susceptibility of myeloma cells to apoptosis through a variety of ways. Reduce the mortality of MM to a certain extent, and may increase the sensitivity and resistance of treatment 33-36. The use of statins is inversely related to the risk of multiple myeloma, and the protective effect increases as the cumulative dose increases 36,37. The present study combined with the established prognostic factors that are routinely used in clinical trials for newly-treated patients, with lipid profiles added, and the performance of the new prognostic model was statistically improved. Therefore, different treatment strategies and active surveillance could be followed in patients in different groups. Compared with the existing staging system, our model has better prediction accuracy and discriminative ability from the perspective of lipid metabolism. This has provided some help for us in studying the lipid profile of MM patients, and for disease prediction and treatment. These indicators have the characteristics of simple detection and dynamic tracking, which are helpful to clinicians in decision-making and formulation of overall individualized treatment management. Although our model suggests that serum lipids increase the prognostic value, the clinical significance of this added value still needs further research.

As mentioned above, the significance of lipid parameters in predicting survival has been confirmed. However, there is no relevant literature that can improve the prognosis of MM after adjusting lipid metabolism while most of the effects of lipid metabolism on tumor prognosis are based on retrospective data analysis to establish predictive models, and only theoretically study the effect of lipid metabolism on prognosis. Retrospective data are mostly cross-sectional studies, short-term follow-up and heterogeneity seemed to be a limitation. Moreover, simple dyslipidemia is difficult to diagnose and rarely treated, and the patients are different from the background population at baseline and have multiple confounding factors. The direction of the relationship between tumor development and changes in blood lipid levels cannot be determined by the results of such studies alone. We look forward to the follow-up study of the interaction between lipid metabolism and tumor prognosis from the molecular mechanism level, which may provide certain help for the whole-process management and prognosis prediction of patients from the perspective of lipid metabolism.

Further research is needed to explore the role of lipids and apolipoproteins in MM. The expression of lipid profile levels in the serum exists for a short time but reflect the overall health status of the patient and liver metabolism function as a whole. This approach is clinically significant and can be used to detect the condition of patients and inform decision-making in the clinic 38.

Whilst our study provided intriguing research findings, it also has several limitations. Firstly, the data collection period was long and it is impossible to systematically compare it with a certain evaluation system as a whole. Secondly, all patient data were collected from a single cancer center which may introduce confounding factors. The sample size was limited and further research is needed to determine if the optimal critical values can be used in a larger range. It is expected that further large-scale, prospective, multicenter studies are needed to validate our findings. Finally, the potential mechanisms of lipid metabolism in the occurrence and development of MM remain to be fully determined.

## Conclusions

We generated a lipid profile-based model to predict the prognosis of MM patients. Lipid profiles are novel prognostic biomarkers that have high accuracy in predicting survival and may inform the future development of precision medicine in patients with MM.

## Supplementary Material

Supplementary figures and table.

## Figures and Tables

**Table 1:** The detailed characteristics of patients and correlation between clinicopathological features and risk score level in patients with multiple myeloma

Variables	All patients, n (%)	Low-risk group, n (%)	High-risk group, n (%)	P-value
Entire cohort	275 (100.0)	148 (53.8)	127 (42.6)	
**Age**				0.275
<65	199 (72.4)	111 (40.4)	87 (31.6)	
≥65	76 (27.6)	37 (13.5)	40 (14.5)	
**Gender**				0.163
female	164 (59.6)	82 (29.8)	82 (29.8)	
male	111 (40.3)	65 (23.6)	46 (16.7)	
**BMI**				0.289
<22	116 (42.1)	58 (21.1)	58 (21.1)	
≥22	154 (56)	87 (31.6)	67 (24.4)	
NA	5 (1.8)	5 (1.8)		
**ALB**				0.004
<30	74 (26.9)	29 (10.5)	45 (16.4)	
≥30	200 (72.7)	117 (42.5)	83 (30.2)	
NA	1 (0.3)			
**SCr**				0.000
<81	128 (46.5)	87 (31.6)	41 (14.9)	
≥81	147 (53.4)	60 (21.8)	87 (31.6)	
**Ca**				
<2.5	213 (77.4)	117 (42.5)	96 (34.9)	0.309
≥2.5	59 (21.4)	28 (10.2)	31 (11.3)	
NA	3 (1.1)			
**ApoB**				0.000
<1.1	147 (53.5)	57 (20.7)	90 (32.7)	
≥1.1	128 (46.5)	13 (4.7)	115 (41.8)	
**ApoA1**				0.000
<1.05	154 (56)	64 (23.3)	90 (32.7)	
≥1.05	121 (44)	83 (30.2)	38 (13.8)	
NA		14 (5.1)		
**ApoB/ApoA1**				0.070
<1.09	97 (35.2)	59 (21.5)	38 (13.8)	
≥1.09	178 (64.7)	88 (32)	90 (32.7)	
**CHO**				0.000
<3.2	90 (32.7)	31 (11.3)	59 (21.5)	
≥3.2	175 (63.6)	113 (41.1)	62 (22.5)	
NA	10 (3.6)	10 (3.6)		
**TG**				0.000
<1.25	142 (51.6)	96 (34.9)	46 (16.7)	
≥1.25	133 (48.3)	51 (18.5)	82 (29.8)	
**HDL**				0.000
<0.9	135 (49)	41 (14.9)	94 (34.2)	
≥0.9	140 (50.9)	108 (39.3)	32 (11.6)	
NA		10 (3.6)		
**LDH**				
<215	227 (82.5)	137 (49.8)	90 (32.7)	0.000
≥215	48 (17.4)	10 (3.6)	38 (13.8)	
**LDL**				0.002
<1.9	125 (45.4)	54 (19.6)	71 (25.8)	
≥1.9	150 (54.5)	93 (33.8)	57 (20.7)	
**ᵝ2MG**				0.000
<5	126 (45.8)	102 (37.1)	24 (8.7)	
≥5	149 (54.1)	45 (16.4)	104 (37.8)	
**ISS stage**				0.000
I	45 (16.3)	37 (13.5)	8 (2.9)	
II	83 (30.1)	59 (21.5)	24 (8.7)	
III	147 (53.4)	51 (18.5)	96 (34.9)	
**DS stage**				0.000
I	55 (16.4)	44 (16)	11 (4)	
II	77 (28)	51 (18.5)	26 (9.5)	
III	143 (52)	52 (18.9)	91 (33.1)	
**Initial therapy**				0.000
Bortezomib based	96 (34.9)	65 (23.6)	31 (11.3)	
Classical drugs-based	179 (65.1)	28 (10.2)	151 (54.9)	
**Treatment response**				0.000
CR	56 (20.3)	38 (13.8)	18 (6.5)	
Less than CR	219 (79.6)	16 (5.8)	203 (73.8)	

Abbreviation: ALB, albumin; SCr, serum creatinine; Ca, calcium; Apo B, apolipoprotein B; Apo A1, apolipoprotein A1; CHO, cholesterin; TG, triglyceride; HDL, high density lipoprotein; LDL, low density lipoprotein; LDH, lactate dehydrogenase; β2MG, β2 microglobin; ISS, International Staging System; DS, Durie-Salmon; CR, complete response.
