# Supplementary material for: A new prediction model integrated serum lipid profile for patients with multiple myeloma
Source: J Cancer. 2022 Mar 14;13(6):1796–807. doi: 10.7150/jca.69321 (PMC8990419; doi:10.7150/jca.69321)
Supplement: Supplementary file 1 — Supplementary figures and table. [file jcav13p1796s1.pdf]

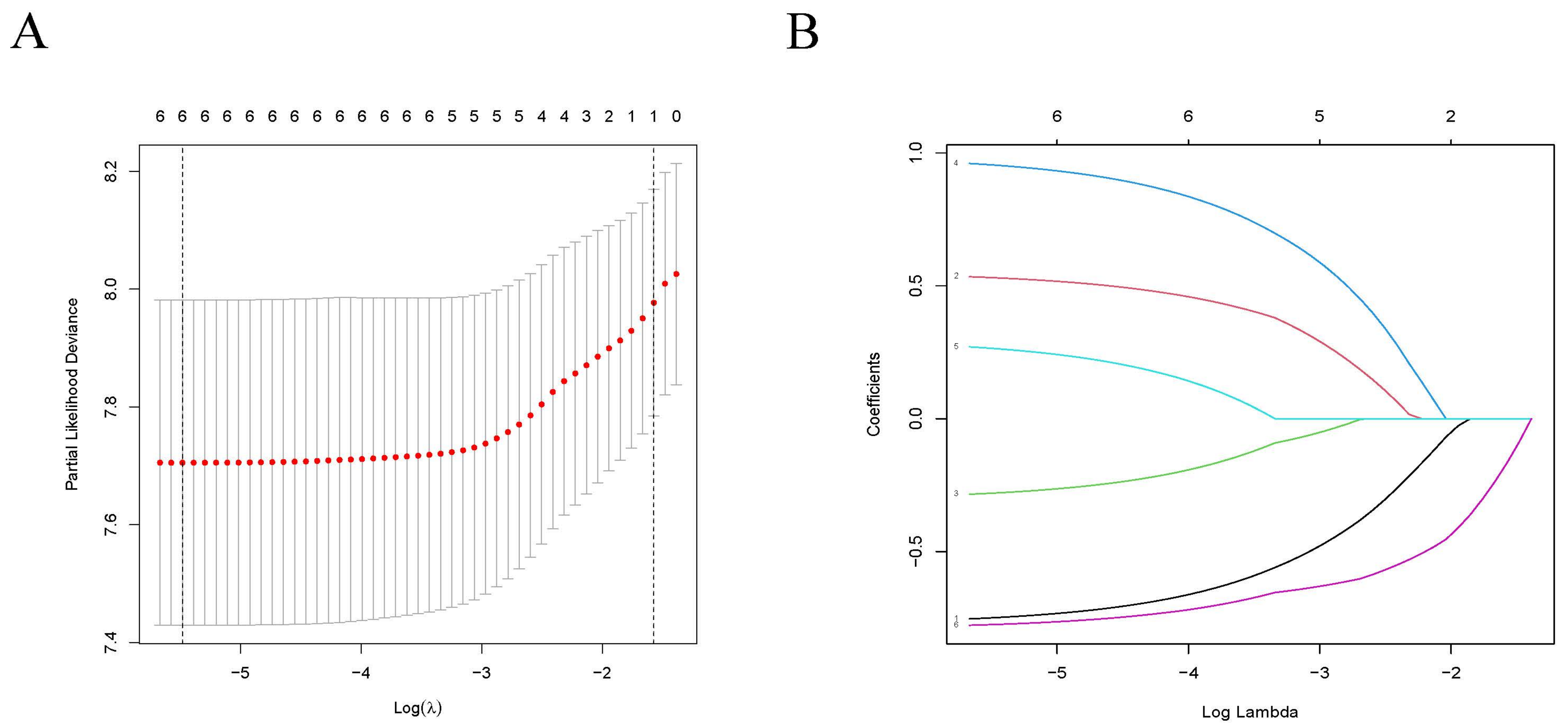

Supplemental Figure 1. Construction of the model for MM. A. 1000 bootstrap replicates by lasso regression analysis for variable selection. B. LASSO coefficients of lipids and apolipoproteins. Each curve represents a lipid and apolipoprotein factor.

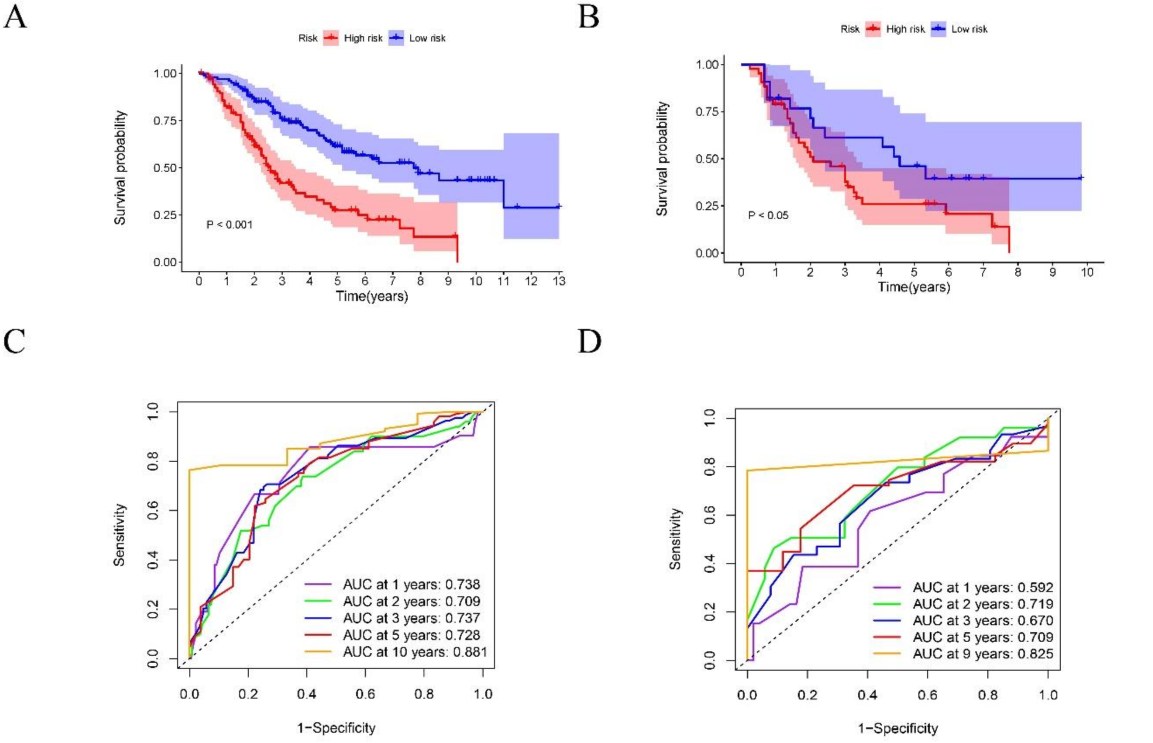

Supplemental Figure 2. Kaplan Meier survival analysis between low-and high-level groups of classical drugs-based(A)and bortezomib-based regimens(B). Time-dependent receiver operating characteristic (ROC) analysis of overall survival (OS) of lipids risk score compared to the classical drugs-based(C)and bortezomib-based regimens(D).

Supplemental Table 1. The baseline characteristics and clinicopathological features of patients with multiple myeloma.

**Variables**	**No. of patients (%)**<br>N=275	**Variables**	**No. of patients (%)**<br>N=275
**age**		<1.9	125(45.4)
<60	137(49.8)	≥1.9	150(54.5)
≥60	138(50.2)	**HDL**	
		<0.9	135(49.1)
**gender**		≥0.9	140(50.9)
male	164(59.6)	**β2MG**	
female	111(40.3)	<5	126(45.8)
**BMI**		≥5	149(54.1)
< 22	91(33.1)	**M protein**	
⩾22	184(66.9)	IgG	144(52)
**ALB**		IgM	2(0.7)
<30	74（26.9）	IgD	1(0.3)
≥30	201（73.1）	NA	61(22.1)
**CREA**		I	45（16.3）
<81	128(46.5)	II	83（30.1）
≥81	147(53.4)	III	147（53.4）
**Ca**		**DS stage**	
<2.5	217(78.9)	I	55（16.4）
≥2.5	58(21.1)	II	77（28）
**ApoB**		III	143（52）
<1.1	205(74.5)	**initial therapy**	
≥1.1	70(25.5)	bortezomib based	96(34.9)
**ApoA1**		classical	179(65.1)

<1.05	157(57.1)	drugs-based **treatment response**	
≥1.05	118(42.9)	CR	56(20.3)
**ApoB/A1**		less than CR	219(79.6)
<1.09	97(35.2)		median(mean±sd)
≥1.09	178(64.7)	ApoB	0.875(11.31±2.115)
**TG**		ApoA1	1.000(9.61±1.760)
<1.25	142(51.6)	ApoB/ApoA1	26.144(44.397±3.265)
≥1.25	133(48.3)	CHO	3.690(3.898±1.022)
**LDH**		TG	1.180(1.486±0.066)
<215	227(82.5)	HDL	0.930(0.994±0.023)
≥215	48(17.4)	LDH	166.000(223.210±23.282)
**CHO**		LDL	2.070(2.310±0.081)
<3.2	101 (36.7)	β2MG	5.610(8.370±0.544)
≥3.2	174(63.3)	ALB	34.950(34.891±0.480)
**LDL**		CREA	84.100(119.710±7.630)

Abbreviation: ALB, albumin; SCr, serum creatinine; Ca, calcium; Apo B, apolipoprotein B; Apo A1, apolipoprotein A1; CHO, cholesterin; TG, triglyceride; HDL, high density lipoprotein; LDL, low density lipoprotein; LDH, lactate dehydrogenase; β2MG, β2 microglobin; ISS, International Staging System; DS, Durie-Salmon; CR, complete response.
